# A comparison of technicques to disimpact the fetal head on a second stage caesearean simulator

**DOI:** 10.1186/s12884-021-04322-2

**Published:** 2022-01-15

**Authors:** Anastasia Martin, Diane Nzelu, Annette Briley, Graham Tydeman, Andrew Shennan

**Affiliations:** 1 Department of Women and Children’s Health, King’s College London, St Thomas’ Hospital, London, UK; 2 Caring Futures Institute, College of Nursing and Health Sciences, Flinders University and King’s College London, London, UK; 3 NHS Fife, Kirkcaldy, KY3 5AH UK

**Keywords:** Fetal head impaction, Full dilation Caesarean section, Caesarean section, Fetal pillow, Tydeman tube

## Abstract

**Background:**

The rate of second stage caesarean section (CS) is rising with associated increases in maternal and neonatal morbidity, which may be related to impaction of the fetal head in the maternal pelvis. In the last 10 years, two devices have been developed to aid disimpaction and reduce these risks: the Fetal Pillow (FP) and the Tydeman Tube (TT). The aim of this study was to determine the distance of upward fetal head elevation achieved on a simulator for second stage CS using these two devices, compared to the established technique of per vaginum digital disimpaction by an assistant.

**Methods:**

We measured elevation of the fetal head achieved with the two devices (TT and FP), compared to digital elevation, on a second stage Caesearean simulator (Desperate Debra ™ set at three levels of severity. Elevation was measured by both a single operator experienced with use of the TT and FP and also multiple assistants with no previous experience of using either device. All measurements were blinded

**Results:**

The trained user achieved greater elevation of the fetal head at both moderate and high levels of severity with the TT (moderate: 30mm vs 12.5mm p<0.001; most severe: 25mm vs 10mm p<0.001) compared to digital elevation. The FP provided comparable elevation to digital at both settings (moderate: 10 vs 12.5mm p=0.149; severe 10 vs 10mm p=0.44).

With untrained users, elevation was also significantly greater with the TT compared to digital elevation (20mm vs 10mm p<0.01). However digital disimpaction was significantly greater than the FP (10mm vs 0mm p<0.0001).

**Conclusion:**

On a simulator, with trained operators, the TT provided greater fetal head elevation than digital elevation and the FP. The FP achieved similar elevation to the digital technique, especially when the user was trained in the procedure.

## Introduction

Over the last two decades, the rate of caesarean section (CS) has risen significantly [1] with CS at full dilatation approximately doubling [2]. A prospective study in a UK hospital in 2019 showed that 17% of births were by emergency CS [3]. Difficulty in delivering the fetal head was found to occur in 20% of emergency CS and 63% at full dilatation [3].

In comparison to CS during the first stage of labour, maternal and neonatal morbidity is higher at full dilatation [4–6]. A more than doubling in rates of both bowel or bladder injuries and extension of the uterine incision, and higher rates of neonatal admissions are reported [4, 5, 7]. These reflect the consequences of prolonged labour and the need for disimpaction of the fetal head from the maternal pelvis prior to delivery through the maternal abdomen [4, 6, 8].

In order to facilitate elevation and disimpaction of the fetal head during a full dilatation CS, a number of techniques have been proposed. In current obstetric practive in the UK the digital ‘push-up’ method is most commonly used, but the reverse breech ‘pull’ method is also described [9, 10]. Alternative methods using vaginal devices have been introduced, aiming to reduce the trauma associated with these techniques. The original device was the Murless blade developed in the 1950s, but there is no evidence it is used in current obstetric practice. The C-Snorkel was developed but found to be ineffective and has been removed from the market. The Fetal Pillow (FP) (Safe Obstetric Systems™) is a balloon device that must be inserted into the vagina prior to surgery. Inflation of the balloon with saline provides upwards elevation and associated disimpaction of the fetal head. A retrospective cohort study concluded that the data to support the use of the FP, at present, is inadequate with no significant benefit of using the fetal pillow [11]. More recently, the Tydeman Tube (TT) has been developed. It is a hollow silicone tube with a cup at the end which aims to maximise force to facilitate disimpaction while minimising pressure on the fetal head [12]. Both the TT and the FP have a greater surface area on the fetal head compared to finger tips, even though the force maybe greater, the risk of fetal head injury is lower. We have quantified this for the TT as per the Vousden et al study [11]. The force that can be applied is fixed for the FP, and is over a large area.

Preliminary data, using a simulator and in clinical practice have demonstrated that, in comparison to the digital ‘push-up’ method, the TT achieved greater fetal head elevation [12].

The aim of the current study was to compare the efficacies of fetal head disimpaction by the commonly practiced digital ‘push-up’ method and these two vaginal devices. The objective of this study was to estimate the distance of upward fetal head elevation achieved using the digital disimpaction compared to both the TT and FP using a simulator-based training session.

## Materials and Methods

This clinical evaluation took place during a simulator-based training session of obstetric staff for the disimpaction of the fetal head during a full dilatation CS at a tertiary London Hospital, where the current practice is digital disimpaction. All clinicians who met the criteria that were present on the day of the simulation session were approached and invited to the training. The fetal head disimpaction methods evaluated were the TT and the FP using digital disimpaction as the comparator using the commercially available simulator Desperate Debra (Adam Rouilly Ltd). The simulator replicates fetal head delivery at full dilatation, allowing for delivery through a transverse suprapubic skin incision. It contains an external screw which allows for manual adjustment of degree of head impaction from minor to severe. A video of the simulator is available online at https://www.youtube.com/watch?v=spTVjcMH-N0.

Two simulation sessions on two separate days took place. As the use of the TT involves co-operation between elevator and surgeon, one session involved mutliple surgeons but a single elevator, who was trained in the use of both devices. The other session involved multiple elevators, who were unfamiliar with either the FP or the TT and a single surgeon. Session one was for training to deliver the impacted head, and session two, training for elevation.

Elevation data were analysed in both sessions. For the first, the single trained operator performed insertion of vaginal devices and digital disimpaction, but multiple operators attempted to deliver the head. The time to delivery was counted from when the TT had been inserted, and when the FP had been inserted and inflated until delivery was achieved. As such the time taken to inflate the FP was not included. The simulator used has been previously described [12]. The force required to achieve certain elevations was measured by applying incremental forces to the axis shaft until the same elevation was achieved. The degree of fetal head impaction was adjusted on the simulator to three different levels of difficulty; mild, moderate and severe. The fetal head position was standardised to a fully flexed, left occipito- transverse on the model for all attempts.

Participants included any obstetric medical staff involved in performing full dilated CS, and ranging from senior house officers to consultants. For each level of fetal head impaction, the operator made three attempts to elevate the fetal head as high out of the pelvis as possible. The first three control measurements, were performed using digital disimpaction. Three attempts for each study device were then performed consecutively in alternating order i.e. three attempts using the TT followed by three attempts using the FP and vice versa. The difficulty levels in which this was done first was also alternated between mild, moderate and severe. An independent observer measured the degree of elevation that the device achieved on each occasion, using a calliper. A wooden calliper was placed next to the simulator where the edge of the calliper was in contact with the flat end of the simulator connected to the fetus. As the fetus’ head is elevated, the flat end pushed the calliper forward in a linear fashion. The observer noted the distance that the calliper moved, reflecting elevation of fetal head. As it was not possible to blind this observer to the technique being evaluated, the scale used a random start number and the elevation calculated post hoc by subtracting this from the final elevation reading. The “surgeons” were not informed of the results. The elevation achieved was stipulated as our primary endpoint, but in addition the force was calculated from this measurement and the time taken to achieve disimpaction was measured by the observer. Each participant was asked to rank the three methods from one to three, ranking their preferred device first.

During the second simulation, each participant made a single attempt to elevate using all three methods. Due to time contraints only the moderate level was used in this exercise. The first attempt was made using digital disimpaction, after this operators alternated between TT or FP first when using the devices. An independent observer measured the degree of elevation achieved by the device using the previously described method to achieve a level of blindness.

### Statistical analysis

Numerical data were expressed as median (Interquartile range [IQR]) and mean with confidence intervals. Between group comparisons were made using Mann-Whitney U and a Kolmogrov-Smirnov test. Statistical significance was achieved with a p-value of <0.01 to account for the multiple comparisons made at each setting of the simulator. We also plotted means (with standard deviations) to show graphically.

Based on the degree and consistency of variation from our previous study using the same methodology, we calculated that at least 20 measurments would give adequate power to show significant differences in elevation between our control group and each study technique [12]. Our primary analysis was differences in elevation between digital and each device using all data from each of day 1 (single participant elevating) and day two (multiple participants elevating). We calculated elevation for each setting seperately on day 1. Force and time were secondary endpoints.

#### Ethics Approval

The clinical evalution project was approved by the Local Clinical Goverance Board at Guy’s and St Thomas’ NHS Trust Foundation Hospital. This project only involved clinical staff and no patient participants.

## Results

**Fig. 1:**
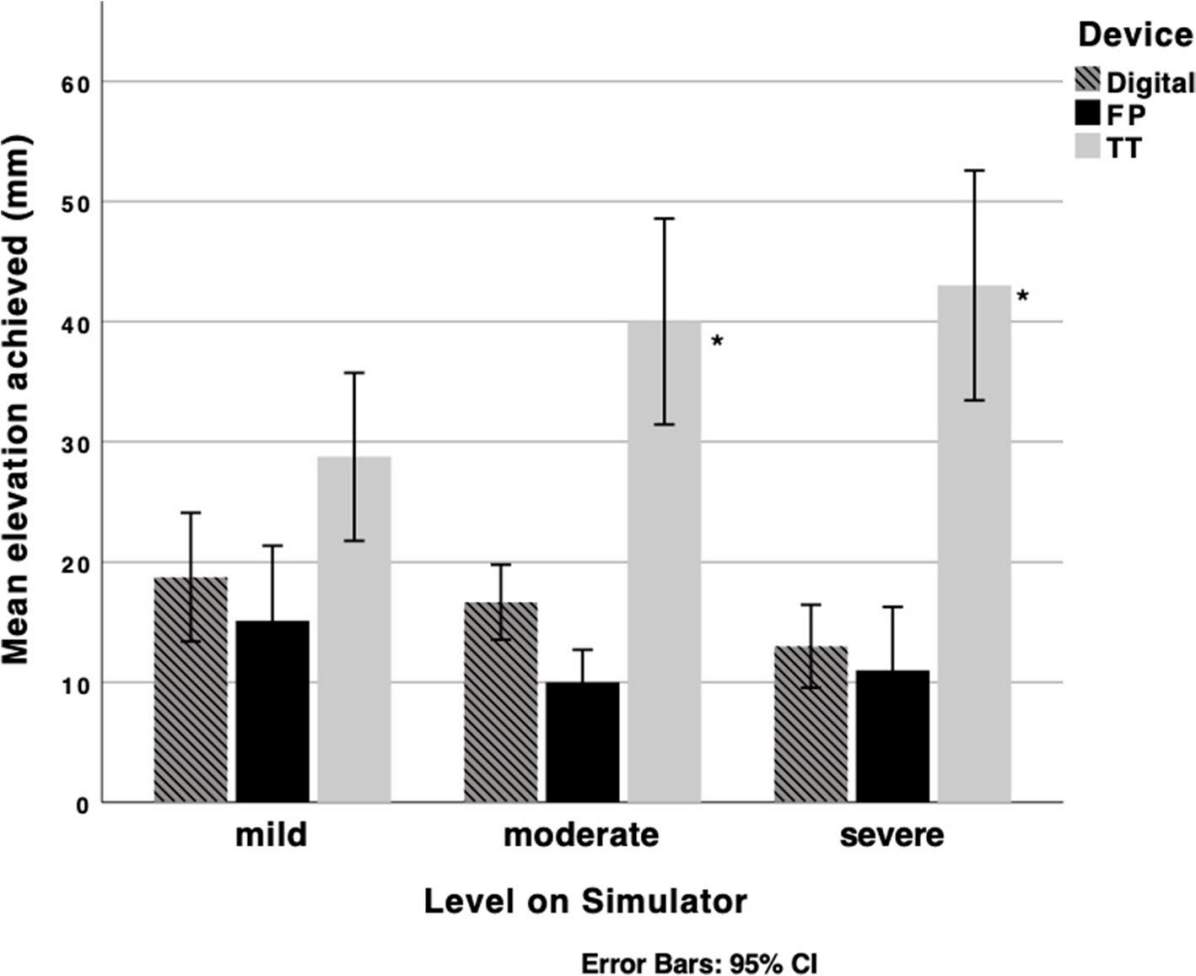
Mean elevation achieved by the Digital disimpaction compared to the 2 study methods with confidence intervals. *p <0.001

For day one, measurements were repeated in 15 training sessions in all those who attended. 90 paired measurements between digital elevation and each test device were obtained (24 for mild, 36 for moderate and 30 for severe settings on the simulator). The median distance of fetal head elevation (mm), along with the calculated force applied (kgf) and the time for the head to be disimpacted at all three levels are presented in Tables 1 and 2, with formal comparison between digital elevation (control) with the two study techniques. The mean distances achieved by all three methods, by simulator setting, are shown in Fig. 1. All recorded measurements were included in the analysis. Multiple surgeons were used to deliver the head with each trio of attempts.

**Table 1:** Elevation (mm) measurements for digital ‘push-up’, Fetal Pillow (FP) and Tydeman Tube (TT) at mild, moderate and severe levels of fetal head impaction

	Device	Distance (mm); Median (IQR)	
		Overall	p-value
Control	Digital	10 (10 – 20)	
1	*Tydeman Tube*	30 (20 – 30)	**<0.001**
2	*Fetal Pillow*	10 (10 – 10)	0.086
		Mild	
Control	Digital	20 (10 – 20)	
1	*Tydeman tube*	20 (20 – 30)	0.1
2	*Fetal Pillow*	10 (10 – 15)	0.20
		Moderate	
Control	Digital	12.5 (10 – 20)	
1	*Tydeman Tube*	30 (30 – 40)	**<0.001**
2	*Fetal Pillow*	10 (10 – 10)	0.149
		Severe	
Control	Digital	10 (10 – 20)	
1	*Tydeman Tube*	25 (25 – 30)	**<0.001**
2	*Fetal Pillow*	10 (10 – 10)	0.44

**Table 2:** Force (kg) and time (s) measurements for digital ‘push-up’, Fetal Pillow and Tydeman Tube at mild, moderate and severe levels of fetal head impaction

	Device	Force (kg): median (IQR)		Time (s): Median (IQR)	
		Overall	*p*-value		*p*-value
Control	Digital	10.0 (8.0 – 11.5)		7.0 (5.0 – 11.5)	
1	*Tydeman Tube*	13.0 (8.8 – 13)	**<0.001**	6.0 (4.3 – 7.8)	0.110
2	*Fetal Pillow*	8.7 (6.0 – 11.0)	0.10	7.0 (5.0 – 12.0)	0.619
		Mild			
Control	Digital	8.0 (6.0 – 8.0)		6.0 (4.0 – 9.0)	
1	*Tydeman Tube*	8.0 (8.0 – 9.0)	0.1	5.0 (4.0 – 7.0)	0.529
2	*Fetal Pillow*	6.0 (6.0 – 7.0)	0.20	6.0 (4.0 – 7.0)	0.467
		Moderate			
Control	Digital	9.4 (8.7 – 12.0)		6.5 (9.0 – 8.25)	
1	*Tydeman Tube*	13.0 (13.0 – 14.0)	**<0.001**	6.0 (4.5 – 8.0)	0.575
2	*Fetal Pillow*	8.7 (8.7 – 8.7)	0.128	8.0 (5.0 – 11.0)	0.331
		Severe			
Control	Digital	11 (11.0 – 13.0)		9.0 (6.3 – 15.0)	
1	*Tydeman Tube*	15.0 (13.0 – 17.0)	**<0.01**	8.5 (6.3 – 25.0)	0.03
2	*Fetal Pillow*	11.0 (11.0 – 11.0)	0.28	15.0 (6.5 – 22.5)	0.642

At the lowest level of difficulty, when the fetal head was minimally impacted, there were no statistical differences in the distance of elevation or force applied between digital disimpaction, and either of the FP and TT. However, at both moderate and high levels of difficulty, where the fetal head was increasingly impacted, the TT achieved greater distance of elevation (p<0.001) than the digital control method, whereas the FP demonstrated similar elevation to the digital tecnique. A post hoc comparison of TT and FP demonstrated that the TT had significantly higher elevation (p<0.001). Similarly, there was a statistically significant increase in force applied by the TT compared to the other digital control in both moderate (p<0.001) and severe (p<0.01) levels, but not for the FP. There were no statistically significant differences in the time taken to achieve disimpaction between any of the methods. No operator failed to deliver the head, even at the most severe setting.

The surgeons were asked their preference after delivering the head using all three methods. Nine preferred the TT as the device that made disimpaction easiest, 4 preferred the FP and 2 felt both devices were equally useful.

**Fig. 2:**
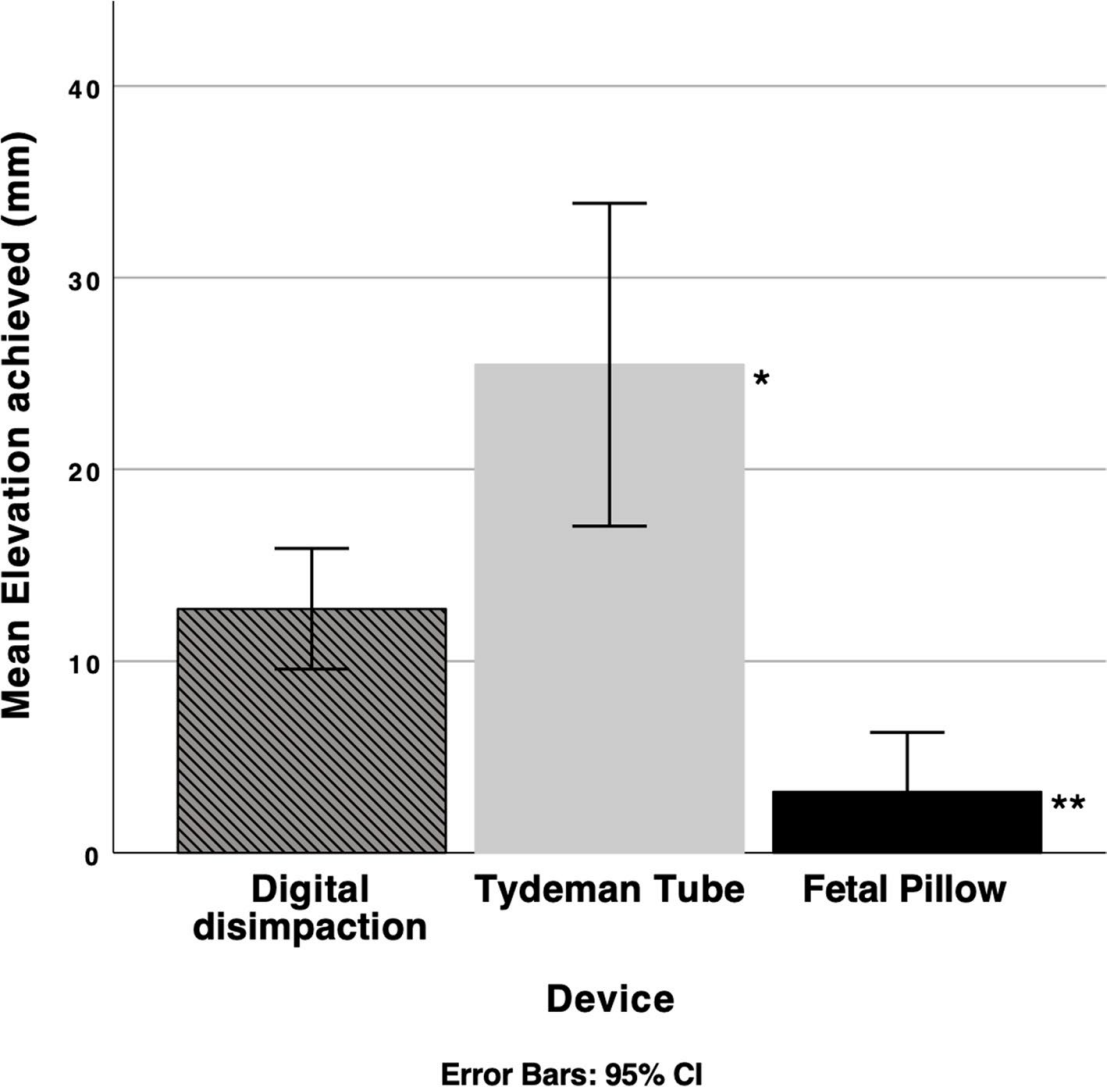
Mean elevation achieved with confidence intervals during second simulation with multiple operators. *p<0.01 **p<0.0001

During the second simulation, 33 paired measurements were obtained, 11 using digital disimpaction, 11 using the TT and 11 using the FP, all at the moderate level. The median distance of fetal head elevation (mm) is presented in Table 3, with formal comparison between the control digital disimpaction method and the two vaginal devices. The TT achieved a significiantly greater elevation than the digitial disimpaction (p<0.01). Digital disimpaction achieved significantly greater elevation than the FP (p<0.0001). The results are shown in Fig. 2. Post-hoc analysis showed that the TT achieved significantly greater elevation than the FP (p<0.0001).

**Table 3:** Median elevation achieved during second simulation, with multiple operators elevating.

	Device	Distance (mm): median (IQR)	
			p-value
Control	Digital	10 (10 – 15)	
1	*Tydeman Tube*	20 (15 – 40)	**<0.01**
2	*Fetal Pillow*	0 (0 – 5)	**<0.0001**

## Discussion

### Principle findings of our study

The findings of this study demonstrate that using a simulator, when compared to the digital ‘push-up’ method, the TT achieves a two to three-fold greater distance of upward fetal head elevation for second stage CS. The FP achieved similar elevation to the digital ‘push-up’ method. The difference in elevation was most prononced at the moderate and severe setting on the simulator. The force achieved to faciliate this elevation was calculated to be significantly greater for the TT. However,previously it has been shown that as the area of contact of the TT is about four fold greater than the area that could be achieved with digits, the pressure applied to the fetal head is lower than the digital technique [11]. The findings were valid for both a single trained “elevator” and for multiple users trying the techniques for the first time. However, the trained “elevator” achieved greater elevation with the TT, suggesting training and experience may assist in the technique. The FP also only elevated on our simulator with the trained “elevator”, suggesting that training in positioning may also be required with the FP, although this may be a result of using the device on a simulator, rather than a real effect.

### Comparison with other studies

The digital ‘push-up’ is one of the most commonly used methods for disimpaction of the fetal head during second stage CS, with more than half UK trainees in obstetrics identifying this as an important method to facilitate birth [13]. This method has reportedly more associated adverse maternal and perinatal outcomes compared to reverse breech extraction [14, 15]. A study of 108 second stage CS demonstrated that disimpaction using the the digital ‘push-up’ was associated with greater intraoperative blood loss (1256±54.3 vs 898±35.2, p<0.001), extension of uterine incision (29.6% vs 11.1%, p<0.05) and a longer postpartum hospital stay (14.5±2.5 vs 11.4±1.2, p<0.001) when compared to the reverse breech ‘pull’ method [14]. Similarly, lower intraoperative blood loss (273±145 vs 403±199, p=0.026), shorter postpartum hospital stay (77.9±19.6 vs 97.8±27.6, p=<0.01) and a higher mean arterial pH (7.24±0.06 vs 7.19±0.09, p=<0.01) were reported in 91 second stage CS incorporating the use of the FP compared with 69 deliveries in which the digital ‘push-up’ method used [16]. The latter study also demonstrated a non-signficant reduction in the rate of uterine incision extensions (20% vs 35%, p=0.061) in the group incorporating the use of the FP [16]. These findings were in agreement with a meta-analysis comparing techniques used for the disimpaction of the fetal head that concluded the digital ‘push-up’ was associated with a significantly higher risk of uterine incision extension [17].

The FP has been evaluated in seven studies, five observational cohorts and two randomised controlled trials with conflicting results in different health systems [11, 18–22]. The use of the FP was associated with improved neonatal outcomes, although a third of babies in the control arm ended up in neonatal intensive ((17.3% vs 33.3%, p=0.025) [18]. In contrast, Seal et al demonstrated significant improvements in maternal outcomes with the use of the FP (n=50) when compared to historical controls with no differences in neonatal outcomes between the two groups [19]. Their findings included a lower rate of uterine incision extensions and shorter operating time and duration of hospital admission associated with the use of the FP. It is important to note that this study did not include any data on their consent process and was undertaken in a very different healthcare system to the National Health System in the UK. The largest observational study to date examining 170 second stage CS in which the FP was used did not report any significant benefit from the use of the FP in preventing maternal or fetal adverse outcomes [11]. Finally, of the two randomised controlled trials, one reported a signficant reduction in the occurrence of major uterine incision extension and a reduction in inoperative blood loss exceeding 1000mls in the FP group whereas the other was underpowered to assess signficiant differences in morbidity [19, 22]. A meta-analysis concluded that the evidence to support the use of the FP is at present inadequate [17].

To our knowledge, there are no other studies that have attempted to objectively measure the distance of upward fetal head elevation achieved by the different methods. One study concluded that on the basis of abdominal palpation before and after insertion of the FP the fetal head was elevated from 0/5 engagement to 2-3/5 engagement, which they estimated as a 4cm fetal head disimpaction [19]. Our study provided an objective estimate of the distance of upward fetal head elevation achieved by the digital ‘push-up’ method in comparison to the FP and the TT. The TT is a newly designed device for the purpose of fetal head disimpaction at second stage CS. Using the same simulator for second stage CS, the TT has previously been demonstrated to upwardly displaced the fetal head, on average, by 3 to 4 cm [12]. Reassuringly, these findings were consistent with our study. Both the single operator during simulation one, and all clinical staff during the second simulation achieved significantly higher elevation with the TT compared to the digital disimpaction. Furthermore, in the study by Vousden et al the TT applied less pressure whilst achieving greater force when compared to digital disimpaction, thereby, minimising the potential for fetal trauma [12]. The greater force required in our study to achieve elevation would not increase trauma to the head given the design of the TT i.e. the pressure is spread over a large area. An additional benefit to the use of the TT is that it does not require prophylactic placement but can be inserted either in anticipation of a deeply impacted head, for example, after a failed instrumental delivery, or following attempt at delivery and can also be used at dilatations less than full when about 20% of impacted heads occur [8]

### Clinical relevance and future research

The reverse breech extraction has been shown to be effective and incur less maternal and neonatal morbidities than the digital elevation. However, when surveyed, less than half of UK trainees in obstetrics felt they would know how to perform the reverse breech ‘pull’ method, when the need arose [13]. Those undertaking second stage CS lack the training to be able to confidently perform a reverse breech ‘pull’ maneovre.

To date, studies that have evaluated devices such as the FP, have a number of limitations. Firstly, they involve small cohorts, and are therefore underpowered to detect clinically important differences in terms of morbidities. Secondly, there is significant heterogeneity both within and between these studies in terms of the type of resouce setting, or the techniques used for fetal head dismpaction in the comparator group [18, 19]. These limitations restrict the generalisability of their findings and likely accounts for the conflicting results.

We have demonstrated that the TT is a potentially effective alternative that provides superior upward head elevation to both the digital ‘push-up’ method and the FP on a simulator for second stage CS. The use of the hand (digital) has the advantage of not requiring preparation time. Both the TT and FP can be inserted preemptively to limit delay but in an emergency there maybe delay in their deployment. The TT is likely to have less delays as only has to be inserted without preparation (inflating the balloon). In view of the increasing rate of second stage CS, an important next step is for a well-designed, adequately powered, randomised controlled trial to evaluate maternal and neonatal outcomes in women undergoing second stage CS, incorporating use of both the FP, the TT or neither device. In clincal practice, sufficient elevation is required to actually achieve delivery, however it may be that if greater elevation is achieved per vaginum, less vigorous effort is required by the surgeon, which may reduce trauma to both mother and child .

### Strengths and limitations

The strengths of this study include obtaining an objective measure of fetal head elevation, achieved using three different methods. The simulator itself is not clinical practice, but has been validated as a realistic alternative for training [12]. The vagina and perineum of the simulator may not represent the optimal environment for the tools to work effectively and comparisons in vivo are required. We chose to compare the disimpaction at a standard fetal position. Even at this setting there were important differences between the devices. They maybe different with more profound disimpaction e.g. with malposition. The outcome of higher elevation is assumed to be of benefit, but clinical studies need to confirm that the use of the TT would translate into better outcomes. The subjective opinion of the operators is consistent with this, but we acknowledge this study involved small numbers.

## Conclusion

In comparison to the digital ‘push-up’ method, the TT provides a greater distance of upward fetal head elevation compared to both the digital ‘push-up’ method and the FP on a simulator for second stage CS. This supports the need for clinical studies to compare these techniques.

## Data Availability

The datasets used and/or analysed during the current study are available from the corresponding author on reasonable request.
